# Differential Modulation of Excitatory and Inhibitory Neurons during Periodic Stimulation

**DOI:** 10.3389/fnins.2016.00062

**Published:** 2016-02-25

**Authors:** Mufti Mahmud, Stefano Vassanelli

**Affiliations:** ^1^NeuroChip Laboratory, Department of Biomedical Sciences, University of PadovaPadova, Italy; ^2^Institute of Information Technology, Jahangirnagar UniversitySavar, Dhaka, Bangladesh

**Keywords:** brain stimulation, transcranial stimulation, periodic stimulation, extracellular stimulation, excitatory and inhibitory neuron, neuronal network, Hodgkin-Huxley model, neurodegenerative diseases

## Abstract

Non-invasive transcranial neuronal stimulation, in addition to deep brain stimulation, is seen as a promising therapeutic and diagnostic approach for an increasing number of neurological diseases such as epilepsy, cluster headaches, depression, specific type of blindness, and other central nervous system disfunctions. Improving its effectiveness and widening its range of use may strongly rely on development of proper stimulation protocols that are tailored to specific brain circuits and that are based on a deep knowledge of different neuron types response to stimulation. To this aim, we have performed a simulation study on the behavior of excitatory and inhibitory neurons subject to sinusoidal stimulation. Due to the intrinsic difference in membrane conductance properties of excitatory and inhibitory neurons, we show that their firing is differentially modulated by the wave parameters. We analyzed the behavior of the two neuronal types for a broad range of stimulus frequency and amplitude and demonstrated that, within a small-world network prototype, parameters tuning allow for a selective enhancement or suppression of the excitation/inhibition ratio.

## 1. Introduction

Non-invasive brain stimulation—i.e., transcranial magentic and current stimulation (Antal and Paulus, 2013; Davis and Koningsbruggen, 2013; Dayan et al., 2013; Paulus, 2014; Shin et al., 2015)—as well as invasive deep brain stimulation (DBS) (McConnell et al., 2012; Miocinovic et al., 2013; Green and Aziz, 2014; Coenen et al., 2015) deeply rely on electrically inducing changes of the neuronal transmembrane potential to modify excitability. Electrical stimulation of neurons has been adopted by many clinicians as means for treatment of a range of neurological disorders. For example, recent reports show its increasing use in Parkinson's disease (Krack et al., 2002; Alon et al., 2012; Obeso et al., 2013; Chou et al., 2015; de Hemptinne et al., 2015), epilepsy (Loddenkemper et al., 2001; Boex et al., 2007; Boon et al., 2009; Fisher et al., 2010; Nelson et al., 2011; Alarcón and Valentín, 2012; Berenyi et al., 2012; Orosz et al., 2014; Salanova et al., 2015), certain types of blindness (Rizzo et al., 2003; Freeman, 2010; Freeman et al., 2010; Antal et al., 2011; Gall et al., 2013), cluster headaches (Grover et al., 2009; Sillay et al., 2010; Matharu and Zrinzo, 2011; Piacentino et al., 2014; Hodaj et al., 2015), depression (Miller and Selman, 2009; Jorge and Robinson, 2011; Rizvi et al., 2011; Anderson et al., 2012; Henn, 2012; Cook et al., 2014; Concerto et al., 2015), obsessive-compulsive disorder (Nuttin et al., 2008; Jiménez-Ponce et al., 2009; Kohl et al., 2014; Grassi et al., 2015; Islam et al., 2015), and other movement disorders like essential tremor (Lozano, 2000; Volkmann and Benecke, 2002; Birdno et al., 2011; Gironell et al., 2014; Lettieri et al., 2015). However, the outcome of the method to many of these applications is reported to be limited due to lack of specificity of the stimulation (Fisher, 2011; Lozano and Lipsman, 2013; Shin et al., 2015). Thus, improvements are to be expected from protocols allowing for selective activation or inhibition of specific target neurons or classes of individual neurons, a strategy that can be broadly referred to as “differential stimulation” and that can be pursued by different approaches.

The “differential stimulation” of neurons can be approached both by means of invasive or non-invasive methods. For example, extracellular microstimulation has been used to selectively activate or inactivate neurons in ganglia using anodic or cathodic currents, respectively (Lu et al., 2008). By an alternative strategy, McIntyre and Grill reported that charge balanced asymmetric biphasic stimuli (McIntyre and Grill, 1999, 2000) can differentially activate neurons or fiber-of-passages by exploiting their difference in voltage thresholds and carefully tuning relevant stimulus parameters, e.g., amplitude, shape, frequency, and localization. They also reported on the effect of stimulus waveform and frequency on central nervous system (CNS) neurons through a detailed computer-based simulation of CNS cells and axons (McIntyre and Grill, 2002), where it was demonstrated that the relative position of the stimulating electrode plays an important role in activating a neuron. Results comparing experimental values and modeling prediction of threshold currents for varied electrode distances have also been reported (Joucla et al., 2012). Intriguingly, sinusoidal stimulation with microelectrodes has emerged as a possible tool to preferentially activate certain retinal cell types (e.g., photoreceptors, bipolar, and ganglion cells) (Freeman et al., 2010) or to induce complex phase-locked firing patterns of cortical pyramidal neurons (Brumberg and Gutkin, 2007).

How neurons are influenced by continuous or alternating electric fields depending on their position with respect to stimulating electrodes, morphology and electrical properties is matter of intense research also in the case of transcranial electrical stimulation approaches, such as the resurgent transcranial current stimulation (TCS) (Ali et al., 2013). For example, studies on transcranial direct current stimulation (tDCS) have shown that exposure to a uniform electric field promotes neuronal bursting and modulates spike timings (Radman et al., 2009). When alternating fields are considered, such as those produced by endogenous oscillations (Fröhlich and McCormick, 2010) and weak external fields (Deans et al., 2007) or by transcranial alterating current stimulation (tACS) (Herrmann et al., 2013; Reato et al., 2013), the general believe is that they can entrain brain oscillations. Furthermore, recent experimental evidence on tACS in humans supports the fascinating idea that excitation/inhibition balance (E/I) can be modulated by tuning the intensity of the stimulation current (Moliadze et al., 2012). The observation could be explained assuming that inhibitory neurons are more sensitive to alternating electrical stimulation and are already activated at low intensities, whereas excitatory neurons would require stronger stimulation. Recently, a similar capability on E/I modulation has been postulated also for tDCS (Krause et al., 2013).

As a matter of fact, understanding how excitatory and inhibitory neurons respond to extracellular electrical stimulation is still an open challenge. A particularly intriguing and clinically relevant aspect is their response to sinusoidal stimuli, such as those employed in tACS, and how it varies by tuning stimulus intensity and over the frequency range (Antal and Paulus, 2013).

This study reports simulation results of excitatory and inhibitory neurons' responses upon sinusoidal stimulation using varied frequencies and amplitudes. We focus on the effect of the extracellular field generated by the stimulus on action potentials firing. We found that it is possible, by careful selection of specific frequencies and amplitudes of the stimulus, to selectively enhance and inhibit either excitatory or inhibitory neurons. We show that the approach can be exploited to differentially modulate neuronal excitability within a network, suggesting its potential usefulness for non-invasive (e.g., tACS Kanai et al., 2008; Zaehle et al., 2010; Liew et al., 2014) as well as invasive brain stimulation (Coenen et al., 2015). Outside the tACS context, the concept can apply to neuroprosthetic devices (e.g., retinal stimulation using multicapacitor / multielectrode array, Eickenscheidt et al., 2012; Ghezzi, 2015; Lewis et al., 2015 and to brain-chip interfacing applications, Vassanelli et al., 2012; Vassanelli, 2014).

## 2. Methods

Single compartment Hodgkin-Huxley (HH) neuron models (Hodgkin and Huxley, 1952) representing two main cortical neuron classes were implemented, the “regular spiking” (RS) excitatory neurons and the “fast spiking” (FS) interneurons (Connors and Gutnick, 1990). The HH model was chosen as it more faithfully describes membrane conductances dynamics with respect to, e.g., an Izhikevich model. This was of primary importance in our context were the neuronal response was investigated across a wide range of frequencies. In the implemented HH model all neurons had two main voltage-dependent ion channels, the Na^+^ and the K^+^, whose conductances, in conjunction with an adjustable leak conductance, were sufficient to generate action potentials. Synaptic interactions were described by conductance-based synaptic currents that implement ionotropic glutamate receptors (AMPA and NMDA) and GABA receptors (GABA_A_) (Destexhe et al., 1994; Börgers et al., 2005). A small-world network of neurons was created by randomly connecting a predefined number of neighboring neurons assigned with a decided connection probability. The following subsections detail the models of the two classes of neurons, their synapses, and the network formation.

### 2.1. Model neuron

Each neuron of both classes (RS and FS) was modeled using single compartment HH type model taken from the literature. There are many variants of kinetic models in the literature to govern the generation of action potentials (Herz et al., 2006) and we selected a model that describes the dynamics of membrane conductances and was previously adopted to match experimental findings (Fröhlich and McCormick, 2010). The constants and parameters used in the model to generate action potentials (see Table 1) were taken from the literature (for RS neuron: Traub et al., 1991 and Mainen et al., 1995; for FS neuron: Wang and Buzsáki, 1996). The membrane potential, *V*, was generated using Equation (1).

(1) $$C_{m}\frac{dV}{dt}=I_{Na}+I_{K}+I_{L}+I_{syn}+I_{app}$$

**Table 1:** **Constants and parameters for individual neuron classes with their units**.

**Parameters**	**Excitatory neuron**	**Inhibitory neuron**	**Unit**
Membrane capacitance, *C*_*m*_	1	1	μF/cm^2^
Sodium reversal potential, *V*_*Na*_	60	55	mV
Potassium reversal potential, *V*_*K*_	−90	−90	mV
Leakage reversal potential, *V*_*L*_	−65	−65	mV
Max. sodium conductance, $\bar{g_{Na}}$	30	35	mS/cm^2^
Max. potassium conductance, $\bar{g_{K}}$	100	9	mS/cm^2^
Max. leakage conductance, $\bar{g_{L}}$	0.1	0.1	mS/cm^2^

Here *I*_*Na*_, *I*_*K*_, *I*_*L*_, *I*_*syn*_, and *I*_*app*_ are the sodium, potassium, leakage, synaptic, and applied currents, respectively and were calculated using Equation (2). All differential equations were solved using second-order Runge-Kutta method (Press et al., 2007) with a step size (*dt*) of 0.05 ms.

(2) $$\begin{array}{l} I_{Na}=\bar{g_{Na}}m^{3}h(V_{Na}-V)\\ I_{K}=\bar{g_{K}}n^{4}(V_{K}-V)\\ I_{L}=\bar{g_{L}}(V_{L}-V)\\ I_{syn}=I_{AMPA}+I_{NMDA}+I_{GABA_{A}}\\ I_{app}=C_{m}\frac{dV_{s}}{dt} \end{array}$$

The symbols $\bar{g_{Na}}$, $\bar{g_{K}}$, and $\bar{g_{L}}$ denote the maximum sodium, potassium, and leakage conductances respectively; *V*_*Na*_, *V*_*K*_, and *V*_*L*_ denote the reversal potentials of those channels; *m*, *h*, and *n* denote the channel gating variables; *I*_*AMPA*_, *I*_*NMDA*_, and *I*_*GAB*_*A*__*A*__ denote the synaptic receptor mediated currents; and *C*_*m*_, *V*, and *V*_*s*_ represent membrane capacitance density, membrane voltage, and applied voltage (i.e., as generated by transcranial or intracranial stimulation), respectively. The parameters and units of the entities are listed in Table 1 and *V* was calculated in mV. For the sake of brevity units will be omitted in the rest of the text.

The channel gating variables, i.e., the activation and inactivation variables for the sodium current (*m* and *h*) and activation variable for the potassium current (*n*) were calculated using Equation (3), where *x* ∈ {*m, h, n*}, and α_*x*_ and β_*x*_ are the voltage dependent transition rates that govern the values taken by activation and inactivation variables.

(3) $$x_{\infty }(V)=\frac{\alpha_{x}(V)}{\alpha_{x}(V)+\beta_{x}(V)}\text{ and }\frac{dx}{dt}=\alpha_{x}(1-x)-\beta_{x}x$$

#### 2.1.1. Excitatory neuron

For the RS neuron, the gating variables were calculated using Equation (3) with *m*_∞_, *h*_∞_, and *n*_∞_ being the initial states of the sodium activation, sodium inactivation, and potassium activation variables, respectively. The voltage dependent transition rates for each of the *m*, *h*, and *n* gating variables were updated using Equations (4, 5, 6), respectively.

(4) $$\begin{array}{l} \alpha_{m}(V)=\frac{0.182(V\;+\;35)}{1-\text{exp}\left[\frac{-(V\;+\;35)}{9}\right]}\text{ and }\beta_{m}(V)=\frac{-0.124(V\;-\;35)}{1-\text{exp}\left[\frac{\left(V\;-\;35\right)}{9}\right]} \end{array}$$

(5) $$\begin{array}{l} \alpha_{h}(V)=\frac{0.024(V\;+\;50)}{1-\text{exp}\left[\frac{-(V\;+\;50)}{5}\right]}\text{ and }\beta_{h}(V)=\frac{-0.0091(V\;-\;75)}{1-\text{exp}\left[\frac{\left(V\;-\;75\right)}{5}\right]} \end{array}$$

(6) $$\begin{array}{l} \alpha_{n}(V)=\frac{0.2(V\;-\;20)}{1-\text{exp}\left[\frac{-(V\;-\;20)}{9}\right]}\text{ and }\beta_{n}(V)=\frac{-0.002(V\;-\;20)}{1-\text{exp}\left[\frac{\left(V\;-\;20\right)}{9}\right]} \end{array}$$

#### 2.1.2. Inhibitory neuron

On the other hand, the voltage dependent transition rates for each of the *m*, *h*, and *n* gating variables were calculated using Equations (7, 8, 9), respectively. The initial states of these gating variables (*m*_∞_, *h*_∞_, and *n*_∞_) were obtained similarly using Equation (3) with their own transition rates.

(7) $$\begin{array}{l} \alpha_{m}(V)=\frac{-0.1(V\;+\;35)}{-1+\text{exp}\left[\frac{-(V\;+\;35)}{10}\right]}\text{ and}\\ \;\beta_{m}(V)=4\text{ exp}\left[\frac{-(V+60)}{18}\right] \end{array}$$

(8) $$\begin{array}{l} \alpha_{h}(V)=0.07\text{ exp}\left[\frac{-(V+58)}{20}\right]\text{ and}\\ \beta_{h}(V)=\frac{1}{1+\text{exp}\left[\frac{-(V\;+\;28)}{10}\right]} \end{array}$$

(9) $$\begin{array}{l} \alpha_{n}(V)=\frac{-0.01(V\;+\;34)}{-1+\text{exp}\left[\frac{-(V\;+\;34)}{10}\right]}\text{ and}\\ \beta_{n}(V)=0.125\text{ exp}\left[\frac{-(V+44)}{80}\right] \end{array}$$

### 2.2. Model synapses

Both GABAergic and glutamatergic synapses were designed to provide inhibition and excitation in the network. The inhibitory synapses were mediated by γ−aminobutyric acid (GABA_A_) receptors, whereas the excitatory synapses were mediated by a combination of α− amino−3− hydroxy−5− methyl−4− isoxazolepropionic acid (AMPA) and N–methyl–D–aspartate (NMDA) receptors (Börgers et al., 2005).

The GABA_A_ mediated synaptic currents were modeled by Equation (10) and summed up for the postsynaptically connecting GABA_A_ mediated synapses.

(10) $$\begin{array}{l} I_{GABA_{A}}=\frac{g}{N_{I}\sum s_{i}(t)(V_{I}-V)}\\ \text{with}\\ \;g=\{\begin{array}{l} g_{IE},\text{ if current neuron is excitatory},\text{and}\\ g_{II},\text{ if current neuron is inhibitory}. \end{array} \end{array}$$

Here *g* is the strength of the synaptic coupling, *N*_*I*_ is the number of presynaptic inhibitory neurons, *V*_*I*_ is the resting potential of the inhibitory neuron (constant value of −70 was used), *V* is the current neuron's membrane potential, and *s*_*i*_ is the gating variable calculated using Equation (11). The *g*_*IE*_ and *g*_*II*_ denote the synaptic coupling strength of inhibitory to excitatory and inhibitory to inhibitory synapses, respectively.

(11) $$\frac{ds}{dt}=\frac{1+tanh(V_{pre}/10)}{2}\frac{1-s}{\tau_{R}}-\frac{s}{\tau_{D}}$$

With τ_*R*_ being the rise time constant (= 0.5 ms), τ_*D*_ being the decay time constant (= 10 ms) and *V*_*pre*_ being the membrane potential of the presynaptic neuron.

The AMPA and NMDA mediated synaptic currents were modeled by Equation (12) and summed up for the postsynaptically connecting AMPA and NMDA mediated synapses based on the synapse type under consideration.

(12) $$\begin{array}{l} I_{AMPA/NMDA}=\frac{g}{N_{E}\sum s_{i}(t)(V_{E}-V)}\\ \text{with}\\ \;g=\{\begin{array}{l} g_{EE},\text{ if current neuron is excitatory},\text{and}\\ g_{EI},\text{ if current neuron is inhibitory}. \end{array} \end{array}$$

Here *N*_*E*_ is the number of presynaptic excitatory neurons with either AMPA or NMDA type synapses, *V*_*E*_ is the resting potential of the excitatory neuron (constant value of −70 was used), *V* is the current neuron's membrane potential, and *s*_*i*_ is the gating variable calculated using either Equation (11) (in case of AMPA mediated synapses) or Equation (13) (in case of NMDA mediated synapses). The *g*_*EE*_ and *g*_*EI*_ denote the synaptic coupling strength of excitatory to excitatory and excitatory to inhibitory synapses, respectively.

(13) $$\frac{ds}{dt}=\frac{1}{1+3.57\text{ exp}(-0.062V_{post})}\frac{1+tanh(V_{pre}/10)}{2}\frac{1-s}{\tau_{R}}-\frac{s}{\tau_{D}}$$

While calculating gating variables for the AMPA receptors, Equation (11) with τ_*R*_ = 0.2 ms and τ_*D*_ = 2 ms was used. On the other hand, the NMDA receptors' gating variables were calculated using Equation (13) with rise time constant τ_*R*_ = 1 ms, decay time constant τ_*D*_ = 100 ms, and *V*_*post*_ as the postsynaptic neuron's membrane potential.

### 2.3. Model small-world network

A small-world (SW) network topology was considered as a prototype model of brain neuronal network (Watts and Strogatz, 1998). As per the definition of SW topology, the network is generated from a ring lattice where each neuron is connecting to *K* neighbors at random with a probability *p* (0 < *p* < 1). Zero probability (*p* = 0) makes the network regular and maximum probability (*p* = 1) makes it a random network (Sun et al., 2011) (see **Figure 4A**).

The neuronal network consisted of RS excitatory neurons (**E**) and FS inhibitory neurons (**I**) at a ratio of 4:1. The **E** and **I** were synaptically connected using SW topology with *K* = 33 neurons and *p* = 0.165. Noteworthy, all the neurons of the SW network were subject to the same applied potential, as it can be reasonably assumed within a small volume of brain tissue that is exposed to an electric field. The choice of single compartment neurons is justified by the fact that the region of the axon hillock (considered to be isopotential with the soma) is by far the most sensitive to external electric stimulation with respect to action potential triggering (Nowak and Bullier, 1998).

The design of synapses are described in the Model Synapses subsection (See Section 2.2). We considered all possible synaptic connections in the network (i.e., **E**→**E**, **E**→**I**, **I**→**E**, and **I**→**I**) with predefined input strengths of arbitrary units (**E**→**E**: 0.1, **E**→**I**: 0.1, **I**→**E**: 0.05, and **I**→**I**: 0.06) to create the connectivity weight matrices without recurrent connectivity. We further used a scaling factor for the excitatory and inhibitory synapses with values 0.03 and 0.06, respectively.

### 2.4. Model background network

The SW network with RS and FS neurons (see Section 2.3) remained at rest without external input. To simulate a background noise input and drive the SW network to spontaneous activity, we used an external neuronal population not exposed to electric stimulation (called “background population,” see **Figure 4B**) consisting of 30 Izhikevich neurons (see Equations 14, 15) (Izhikevich, 2003) randomly connecting to 50% neurons in the target population through AMPA mediated synapses (see Equations 12, 11). In this case, Izhikevich neurons were preferred to HH neurons because computationally favorable and considering that they solely represented a source of spikes.

(14) $$\begin{array}{l} \frac{dv}{dt}=0.04v^{2}+5v+140-u+I\\ \frac{du}{dt}=a(bv-u) \end{array}$$

with an after-spike resetting function defined by Equation (15).

(15) $$\text{if }v\geq 30\text{ mV, then}\{\begin{array}{l} v\leftarrow c\\ u\leftarrow u+d \end{array}$$

Here, *v*, *u*, *t*, and *I* are membrane potential, membrane recovery variable representing the Na^+^ and K^+^ channel kinetics, time, and injected current respectively. *a* is the time scale of *u*, *b* is the sensitivity of *u*, *c* is the after-spike resetting value of *v*, and *d* is the after-spike resetting value of *u*. The values used for *a*, *b*, *c*, and *d* are 0.1, 0.2, −65, and 2 respectively. The values of *v* and *c* are expressed in mV, and *t* in ms.

Each of the neurons in the background population constantly received a zero-mean Gaussian noise (*I* in Equations 14) with a variance of 4.6 that generated enough AMPA mediated excitation to activate the target population which then maintained a steady firing pattern.

## 3. Results and discussion

**Figure 1:**
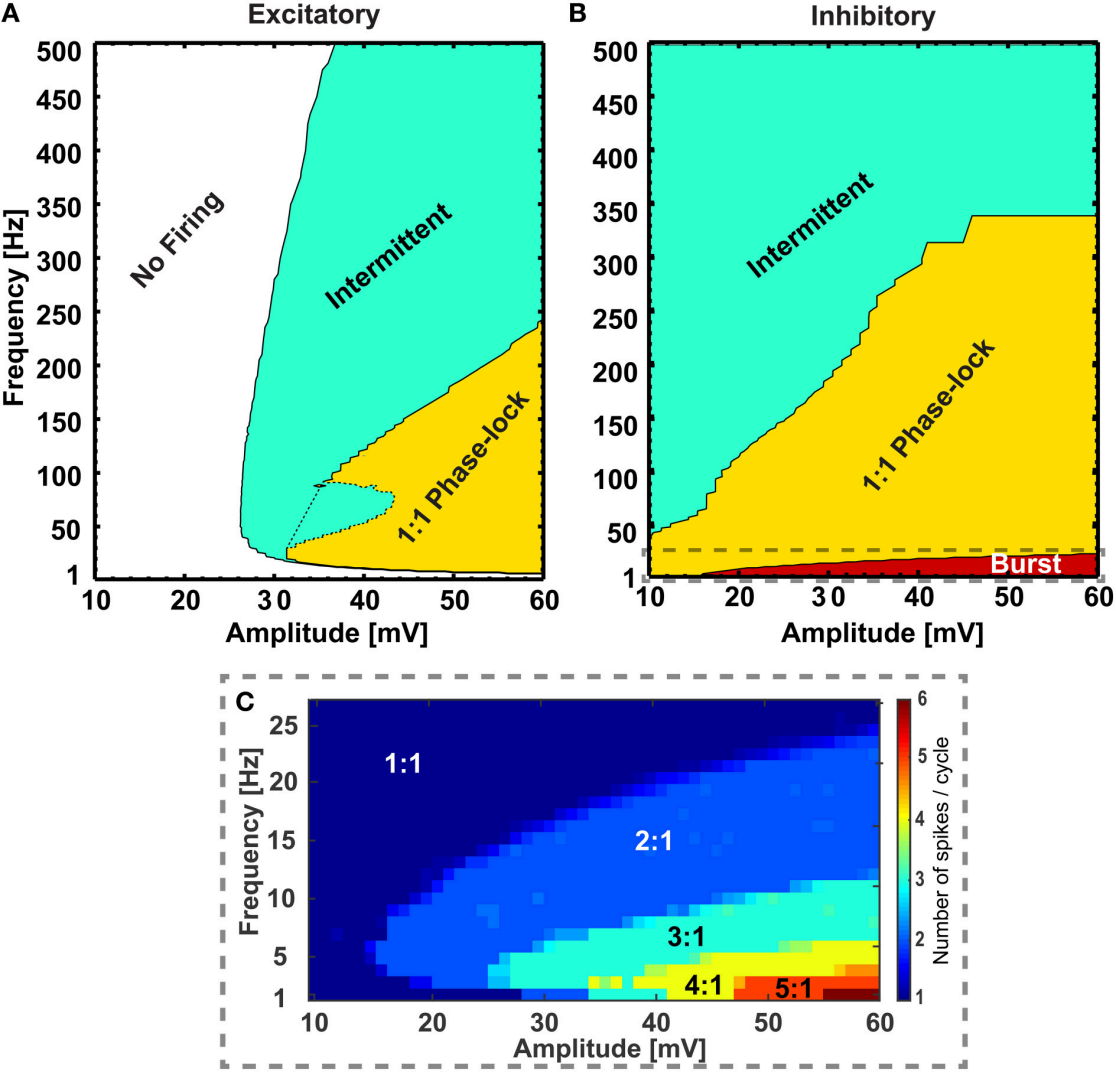
**Different firing regimes during sinusoidal stimulation**. Excitatory **(A)** and inhibitory **(B)** neurons show different firing behaviors upon sinusoidal stimulation with different frequency and amplitude. The excitatory neurons do not respond to a range of amplitudes of sinusoidal stimulations (10− ~ 26 mV, “No Firing” region) and at higher amplitudes (>26 mV) with frequencies greater than ~ 10 Hz they show phase lock (“1:1 Phase-lock” region) and intermittent (“Intermittent” region) firing behavior. Also, at a range of frequencies and amplitudes the excitatory neuron switches between “1:1 Phase-lock” and “Intermittent” firing [the dotted region in **(A)**, referred as “Knee”]. On the other hand, the inhibitory neurons show bursting behavior in low frequencies [“Burst” region, zoomed in **(C)**], with gradual transition to phase-lock (“1:1 Phase-lock” region) and intermittent (“Intermittent” region) firing behavior with increasing amplitude and frequencies of the sinusoidal stimulations. In the burst region, the inhibitory neuron emits a varied number of action-potentials per cycle of stimulation **(C)** starting from 2:1 to 6:1 for the explored range of frequency and amplitude.

We first examined how the individual RS excitatory and FS inhibitory neurons respond to sinusoidal modulation of their transmembrane potential, e.g., as a result of extracellular stimulation with alternating current. We assessed different frequencies and strengths and created maps of firing patterns for the two types of neurons (see Figure 1). Maps show how the amplitude-frequency relationship affects the neuronal firing. Four possible modes were found: (i) non-firing, (ii) phase-lock firing with one action potential per peak (i.e., following a 1:1 relation), (iii) an intermediate condition where peaks of the sinusoidal modulation were not all associated to an action potential (i.e., intermittent firing in Figure 1) and (iv) bursting (i.e., with multiple action potentials per peak). Interestingly, excitatory neurons displayed the first three modes of response (Figure 1A): the no firing mode in the very low frequency range and for low stimulation amplitudes, the phase-locked behavior in the intermediate frequency range and for high amplitudes, and the intermittent firing in between. Conversely, inhibitory neurons were characterized by either bursting (Figure 1C), phase-locked or intermittent behavior when moving from the low to the high frequency range (Figure 1B).

### 3.1. Differential modulation of single neurons

Due to intrinsic differences in the membrane properties between the two classes of neurons, they exhibit variation in their firing patterns while subject to the same periodic modulation as seen in Figure 1. After simulating the firing behavior of neurons from both classes for a large range of stimulating sinusoidal waveforms (frequency range: 1 to 500 Hz, and amplitude range: 10 to 60 mV), invariant to background perturbations (i.e., with or without sub-threshold background inputs to the single neurons), the RS neurons remained silent for a range of amplitudes and frequencies of the input signals (10 ≤ amplitude ≤ 26 mV, 1 ≤ frequency ≤ 10 Hz), but in the same range the FS neurons exhibited firing patterns that varied from bursting to 1:1 phase lock to intermittent, depending on input signal's amplitude and frequency. Furthermore, though the RS neurons responded to input signals with amplitude > 26 mV and frequency > 10 Hz by firing action potentials, the firing pattern was irregular for the range (26 ≤ amplitude ≤ 44 mV, 10 ≤ frequency ≤ 80 Hz), that is the pattern was switching between intermittent and 1:1 phase lock depending on specific amplitude-frequency combinations (dotted area in Figure 1A). On the contrary, in this range of stimulation, the FS neurons show steady progression in firing patterns (i.e., either “1:1 phase lock to intermittent” or “burst to 1:1 phase lock to intermittent”) with increasing stimulus strength and frequency.

**Figure 2:**
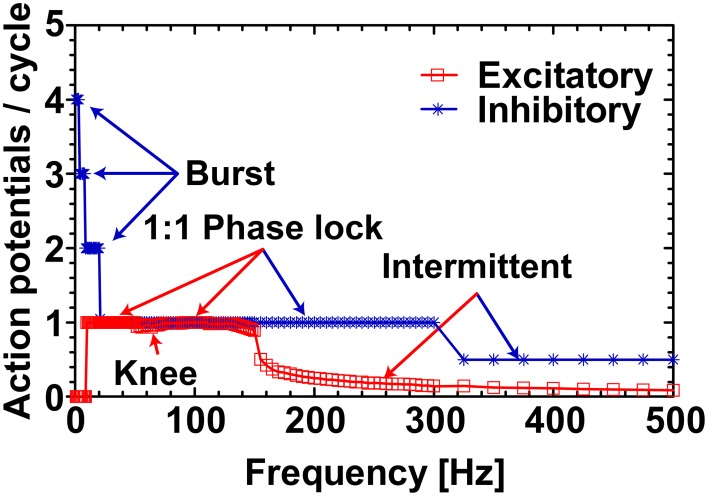
**Excitatory and inhibitory neurons response profile at 38 mV**. The response profile of excitatory and inhibitory neurons as a function of number of spikes generated per cycle of sinusoidal stimulation at 38 mV. The applied frequency was from 1 to 500 Hz with a step of 1 Hz for 1–50 Hz, 2 Hz for 51–150 Hz, 5 Hz for 151–300 Hz, and 25 Hz for 301–500 Hz. The firing behavior outlined (“Burst,” “1:1 Phase lock,” and “Intermittent”) are in complete agreement with the reference map shown in Figure 1. The “Knee” corresponds to the switching behavior (i.e., from 1:1 phase-lock to intermittent and back to 1:1 phase-lock) of the excitatory neuron in the frequency range ~50− ~90 Hz as noticed in Figure 1.

To exemplify the firing behavior mapped in Figure 1, individual neuronal classes were stimulated at 38 mV for the whole frequency range (1 − 500 Hz). As expected, when the number of elicited action potentials per sinusoidal cycle was plotted against the applied frequencies, the individual neuronal classes showed different firing patterns within given frequency ranges Figure 2. The inhibitory neuron (blue circled line) showed bursting behavior (with 4:1, 3:1, and 2:1 action potentials per cycle ratio) for input signals up to 20 Hz and the excitatory neuron (red squared line) remained silent for the first 10 Hz. However, during the subsequent range of intermediate frequencies (20− ~300 Hz), while the inhibitory neuron fired 1:1 phase-locked action potentials, the excitatory neuron first fired in a 1:1 phase-locked mode (10− ~110 Hz) with a short knee-shaped interval to an intermittent state (~50− ~90 Hz), and then stably reverted to an intermittent regime. In fact, for the frequencies above 300 Hz both the neuronal classes showed intermittent firing in agreement with the reference maps.

**Figure 3:**
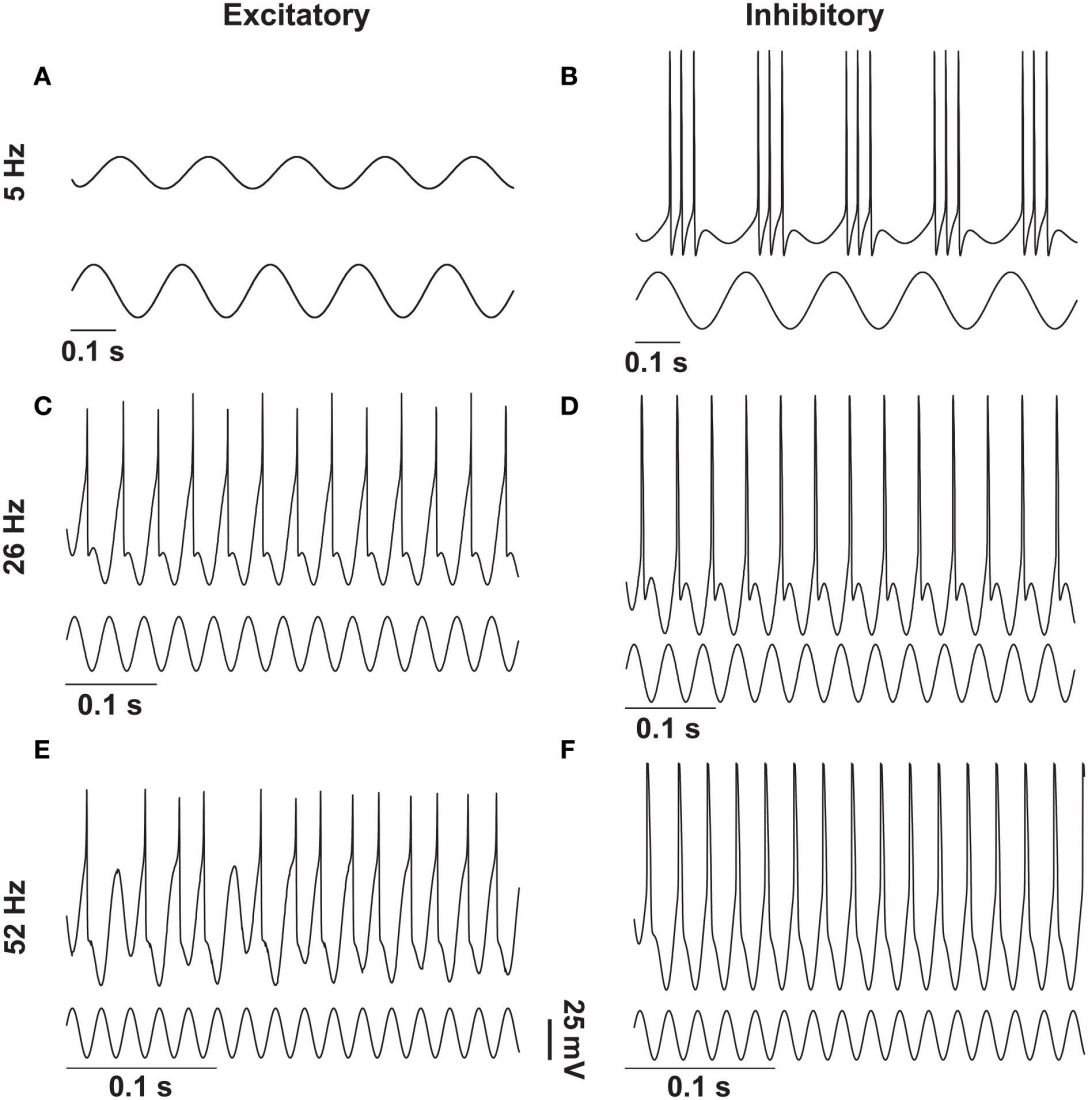
**Response of excitatory and inhibitory neurons to different frequencies of sinusoidal stimulations**. Stimulating resting state excitatory and inhibitory neurons with different frequencies (5 Hz, 26 Hz, and 52 Hz) of sinusoids of 38 mV elicited different firing patterns as outlined in Figure 1. In case of the excitatory neuron 5 Hz stimulation was not enough to elicit action potentials **(A)**, whereas the inhibitory neuron produced bursting activity **(B)**. For 26 Hz action potentials were elicited in both types of neurons in a phase-locked fashion **(C,D)**, and for 52 Hz the excitatory neuron moved to the intermittent region **(E)** and the inhibitory neuron still showed the phase-locked firing **(F)**.

To clearly visualize the differential responses of the two neuronal types to sinusoidal stimulation, we selected three representative frequencies of sinusoidal input signals (5, 26, and 52 Hz) from Figure 2. As seen in Figure 3, at 5 Hz, the inhibitory neuron fires bursts of action potentials (Figure 3B) but the excitatory neuron remains silent (Figure 3A); at 26 Hz, both types of neurons fire action potentials 1:1 phase locked to the stimulation signals (Figure 3C,D); and at 52 Hz, the excitatory neuron exhibits intermittent firing (Figure 3E) while the inhibitory neuron still fires in phase to the input signal (Figure 3F).

### 3.2. Differential modulation of neuronal network

**Figure 4:**
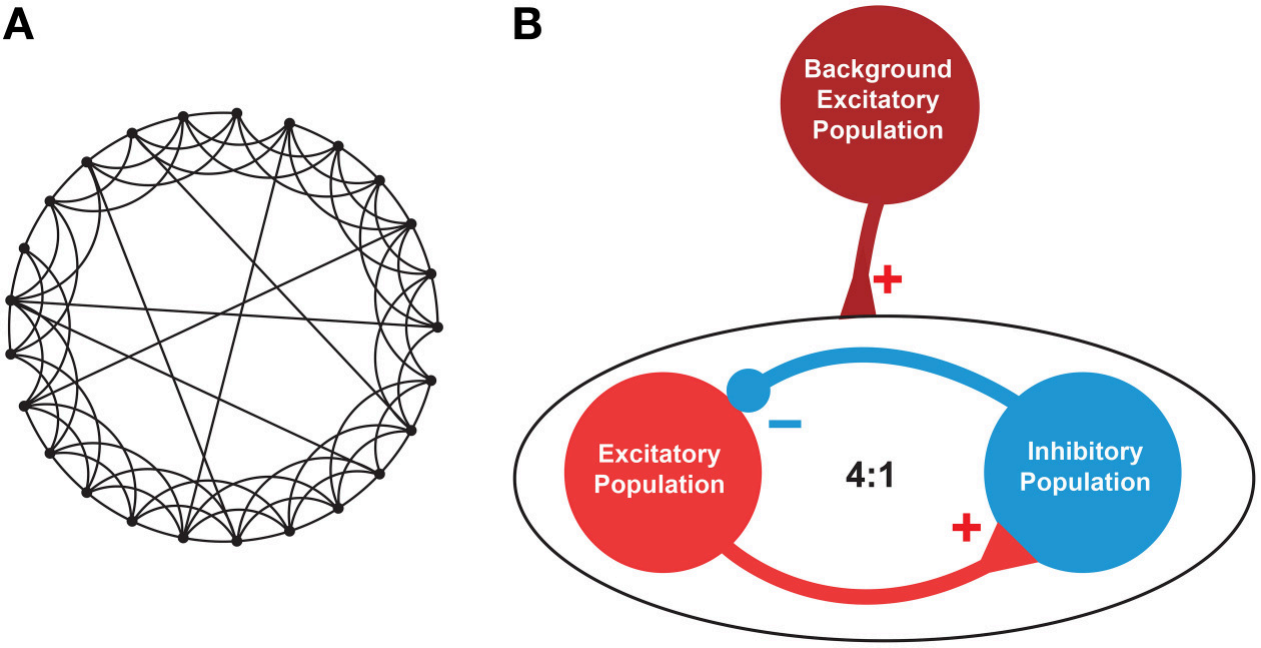
**Architecture of the neuronal network**. **(A)**. An instance of a representative small-world network with 25 nodes out of which 6 (*K*) nodes were linked via random rewiring with probability *p* = 0.24. **(B)**. A network consisting of excitatory (**E**) and inhibitory (**I**) neurons at 4:1 ratio was created with **E**→**E**, **E**→**I**, **I**→**E**, and **I**→**I** synaptic connectivity. To mimic the background activity or driving force, another population of excitatory neurons was used (maroon circle). This background population provided excitatory inputs to 50% of the excitatory (red circle) and inhibitory (blue circle) neurons to maintain a stable firing pattern. The excitatory-inhibitory population consisted of total *N* (= 200, *E* = 160, *I* = 40) neurons, each randomly connected to other *K* (= 33) neurons with *p* (= 0.165) connection probability using undirected edges creating a small-world network as described in Watts and Strogatz (1998).

We further investigated the effect of differential sinusoidal stimulation on neurons forming a neuronal network with SW topology (Figure 4A), a condition more closely resembling real brain circuits with respect to isolated neurons (Bullmore and Sporns, 2012). We assessed two distinct network conditions: (i) when the SW network was silent, i.e., without any external supply except the test inputs; and (ii) when the SW network was driven to be spontaneously active.

**Figure 5:**
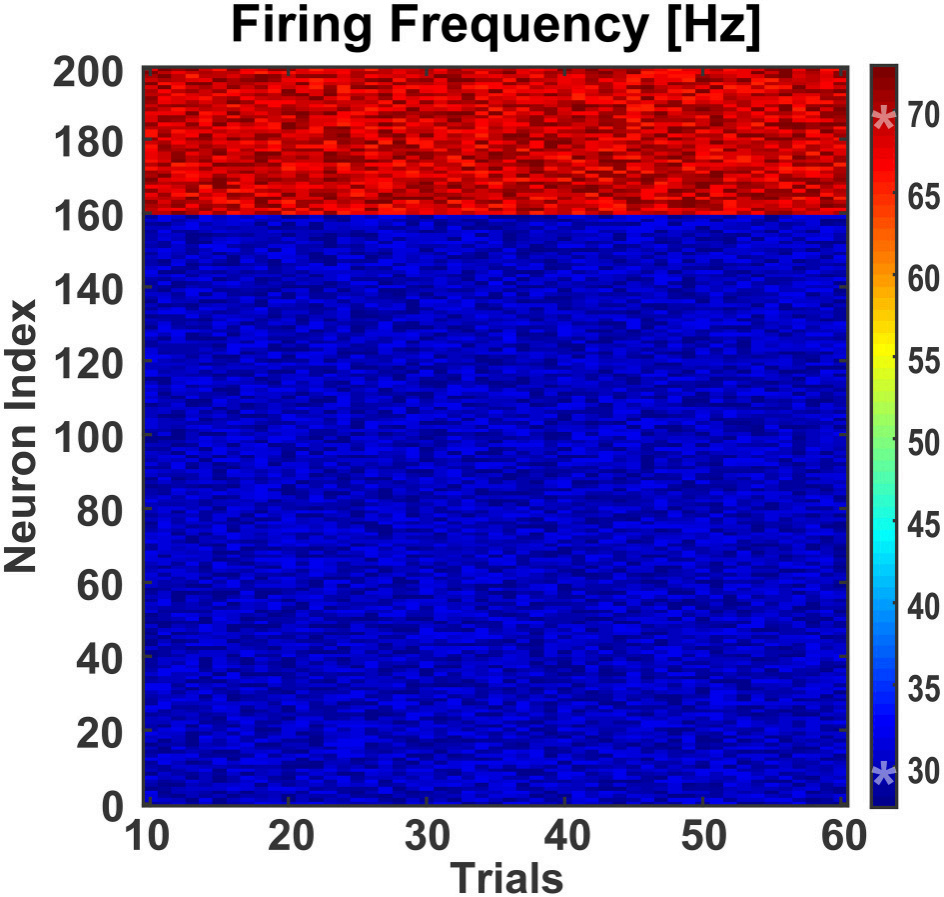
**Effect of background activity on network**. To test the effect of differential modulation of excitatory and inhibitory neurons, the small-world network was fed with excitatory synaptic inputs from a background population consisting of 30 Izhikevich neurons. These neurons were stimulated with a randomly generated zero-mean Gaussian noise of variance 4.6. This generated enough excitatory synaptic conductance to activate the target population of excitatory and inhibitory neurons. Over 60 trials, the mean firing frequencies of the excitatory and inhibitory neurons were 30.00 Hz (±0.11 Hz) and 68.98 Hz (±0.27 Hz), respectively. The two asterisks (‘*’) on the colorbar represent the mean firing frequencies.

The latter condition was achieved by adding an external “background” neuronal population driving the SW network to a basal activity regime (Figure 4B). The background neuronal population was activated by perturbing it with zero-mean Gaussian noise, which caused spikes uniformly distributed in the population. 50% neurons from both classes in the SW network received AMPA mediated excitatory synaptic inputs from the background population which were enough to drive the SW network (see Figure 5) and generate excitatory and inhibitory synaptic conductances comparable to those of a real biological network (Guillamon et al., 2006).

**Figure 6:**
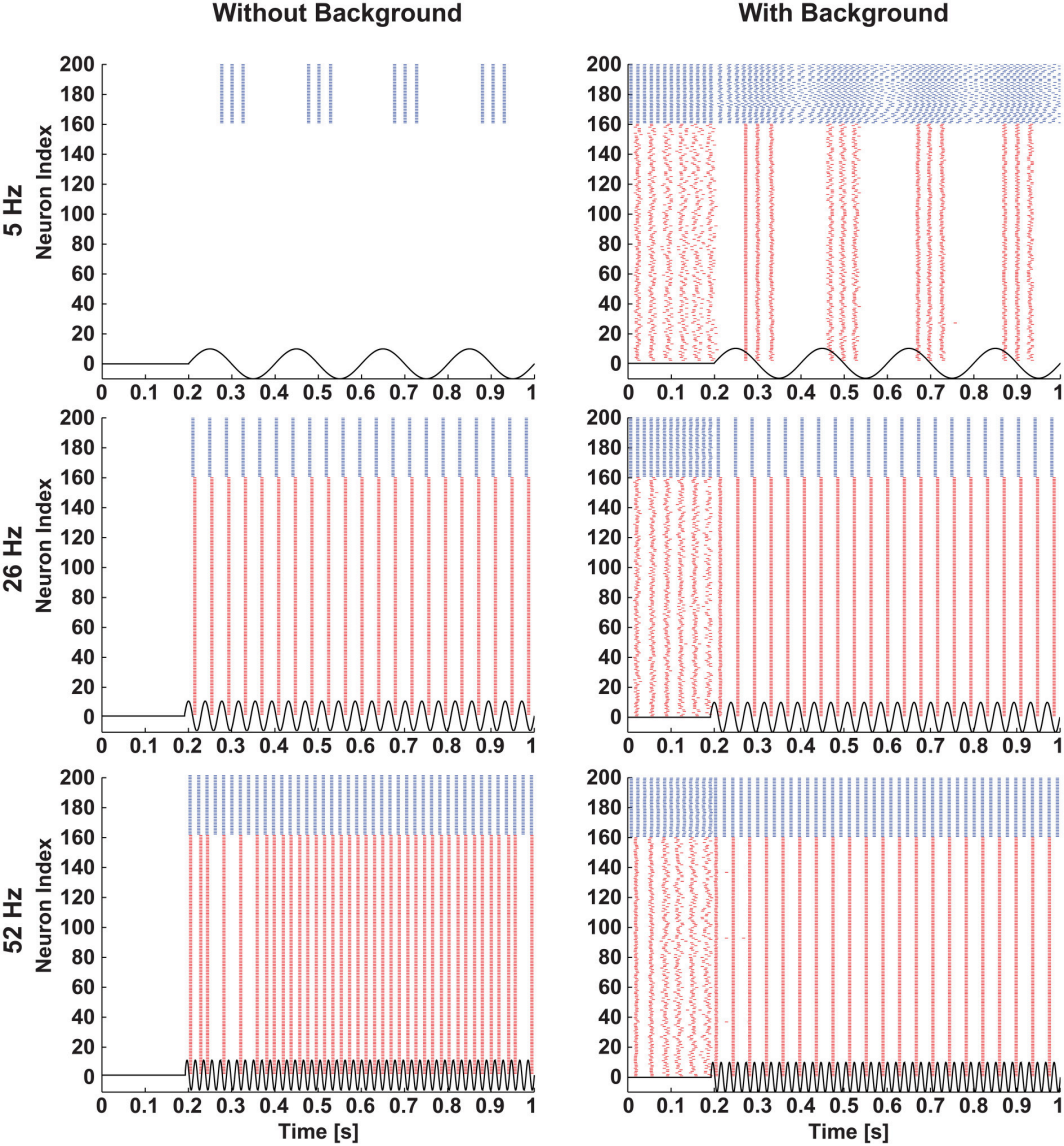
**Selective modulation of excitatory and inhibitory neurons in a network**. A small-world network of excitatory and inhibitory neurons was stimulated with three different frequencies of sinusoids (5 Hz: top row, 26 Hz: middle row, and 52 Hz: bottom row) of 38 mV amplitude when the network was silent (left column) and activated by background excitatory synaptic inputs (right column). Raster plots of the network illustrate that when stimulated with sinusoids, the silent network showed similar firing patterns as in single neurons (see Figure 2), and the spontaneously active network showed selective modulation of excitatory and inhibitory neurons. The blue and red colors represent activities of inhibitory and excitatory neurons, respectively.

**Figure 7:**
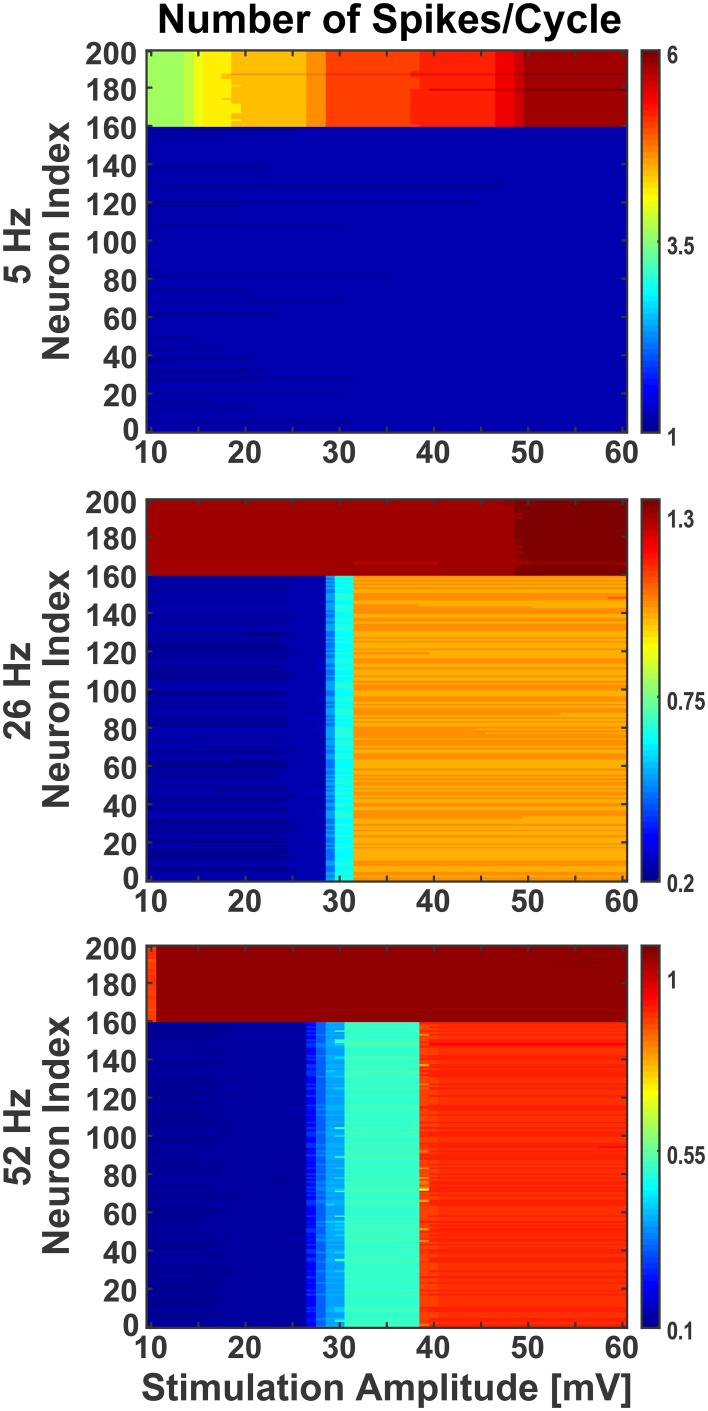
**Effect of stimulation amplitude on active network**. The network was stimulated with varied amplitude of sinusoids (10 mV to 60 mV with step of 1) at three different frequencies (5 Hz, 26 Hz, and 52 Hz), as in Figure 6, when the network was activated by synaptic inputs from the background population. Even during the presence of background activity, stimulating the network with different amplitudes of sinusoids, the individual neurons' firing behavior was comparable to the ones noticed at 38 mV. The Y-axis shows the neurons index where 1 to 160 are excitatory and 161 to 200 are inhibitory. The colorbar shows the number of spikes per cycle.

In both network conditions (i.e., silent and spontaneously active) the individual neurons belonging to different classes were differentially modulated (see Figures 6, 7). In the spontaneously active network, differential modulation was forcing the spontaneously active SW network to a highly synchronized state (Figure 6, right column). This phenomenon can be attributed to the modulation of synaptic coupling in the SW network (Breakspear et al., 2003), and selective amplification of cortical cells' responses at preferred frequencies by intra-network inputs from similarly tuned neurons (Liu et al., 2007; Rotstein and Nadim, 2014). Conversely, in the case of the silent network condition, the modulation (Figure 6, left column) closely matched the single-neuron reference map (see Figure 1).

Moreover, in response to sinusoidal stimulation with given amplitudes and frequencies, the neurons exhibit either an increase or a decrease in their spiking rates with respect to background activity. The inhibitory neurons in the network were found to be more susceptible to sinusoids at lower intensities, matching qualitatively previous experimental observations (Moliadze et al., 2012), and frequencies (Reato et al., 2013). As seen in Figure 7, at low frequency (i.e., 5 Hz) with increasing intensity, the inhibitory neurons show frequent change in action potential firing rate (indicated by color stripes in figure) compared to excitatory neurons which fire steadily (indicated by uniform color in figure). The reverse happens at higher frequencies (i.e., 26 and 52 Hz), where the excitatory neurons show an increasing enhancement of firing rates (indicated by color stripes in figure), while the inhibitory ones fire invariably (indicated by uniform color in figure). This differential activation of excitatory and inhibitory neurons gives rise to a change of the excitation/inhibition ratio (E/I) in the network which is dependent form the amplitude and frequency of the stimulus, and that may represent a mechanism behind experimental and clinical observations during tACS (Antal and Paulus, 2013; Herrmann et al., 2013).

**Figure 8:**
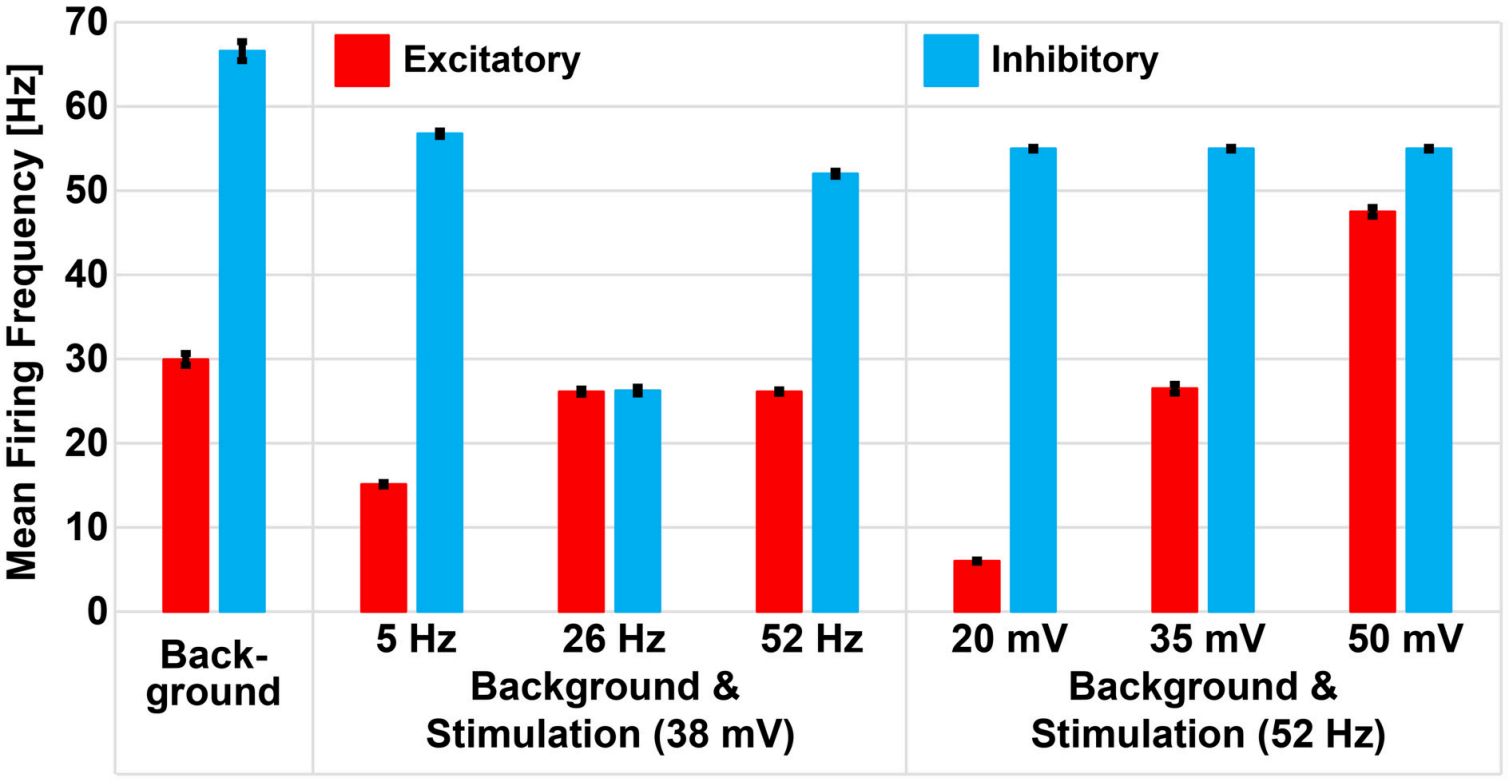
**Repetitive stimulation changes mean firing rate of excitatory and inhibitory neurons in presence of background activity**. Excitatory and inhibitory neurons of the small-world network had mean firing frequencies of 29.94 Hz (± 0.7 Hz) and 66.56 Hz (± 1.12 Hz), respectively, as a result of excitation received from the excitatory background population. Sinusoidal stimulation at 38 mV selectively modulated the mean firing rate of excitatory and inhibitory neurons to 15.12 Hz (± 0.2 Hz) and 56.75 Hz (± 0.32 Hz) during 5 Hz, 26.12 Hz (± 0.1 Hz) and 26.24 Hz (± 0.1 Hz) during 26 Hz, and 26.12 Hz (± 0.14 Hz) and 51.99 Hz (± 0.1 Hz) during 52 Hz stimulation. However, fixing the stimulation frequency at 52 Hz and varying the amplitudes, we noticed gradual enhancement of excitatory neuron only (i.e., from 6.02 ± 0.12 Hz to 26.47 ± 0.51 Hz to 47.47 ± 0.52 Hz) while the inhibitory neuron remained unchanged (54.97 ± 0.16 Hz).

Dysregulation of E/I has been associated to many CNS disorders (Eichler and Meier, 2008), characterized by inefficient information exchange in brain regions. This inefficacy could be caused by loss of homeostatic control of excitation and inhibition (Krause et al., 2013), making it crucial to find therapeutic approaches to restore physiological E/I. To this aim, also on the basis of our results, the E/I may be modulated by finely tuning the amplitude and frequency of sinusoidal stimulation. The concept is evidenced in Figure 8 were we show how, by changing stimulation parameters, the average activity of excitatory and inhibitory neurons in a spontaneoulsy active network can be tuned modulating, in turn, the E/I.

## 4. Conclusion

We provide evidence that, leveraging the different properties of voltage-dependent membrane conductances in excitatory and inhibitory neurons, sinusoidal stimuli can be used to differentially modulate their firing. In particular, basing on simulations of a network of excitatory and inhibitory neurons exposed to a sinusoidal modulation of the extracellular potential, we showed that sinusoidal stimulation could modulate the E/I. In practice, all electrical stimulation methods adopted in the experimental and clinical context and causing sinusoidal voltage changes in the extracellular fluid of the brain tissue could be suitable for the purpose. These include implanted electrodes, such as in DBS, and transcranial non-invasive stimulation approaches such as tACS. However, further elaboration will be necessary to assess the real potential of the approach in clinics. First of all, an unknown contribution will exist from fibers stimulation by the electric field (Roth and Basser, 1990; Herrmann et al., 2013). Second, synaptic plasticity phenomena may also influence network dynamics upon sinusoidal stimulation, as proposed by Antal and Paulus (2013); Zaehle et al. (2010). Finally, it will be crucial to precisely estimate the transmembrane potential in neurons during tACS, taking into account the impedence of the neuronal membrane and its shunting influence at higher frequencies. In fact, despite technical advances to strengthen stimulation (Herrmann et al., 2013), the transmembrane potential modulation caused by tACS may turn out to be too weak to control E/I for clinical usage. Despite these unknowns, and in future perspective, differential sinusoidal stimulation may prove to be a versatile approach in clinics to restore physiological balance between excitation and inhibition in a number of neurological disorders.

## Author contributions

SV conceived the basic idea of differential sinusoidal stimulation; MM developed the model and wrote the code to assess the differential sinusoidal stimulation concept. MM and SV wrote the manuscript. Both authors have contributed to, seen and approved the final manuscript.

## Funding

Financial support from the 7th Framework Programme of the European Commission through “CyberRat” (http://www.vassanellilab.eu/projects/cyberrat/, GA no. 216528) and “RAMP” projects (www.rampproject.eu, GA no. 612058) are acknowledged.

### Conflict of interest statement

The authors declare that the research was conducted in the absence of any commercial or financial relationships that could be construed as a potential conflict of interest.
